# Case Series: Endoscopic ultrasound-directed transgastric ERCP in liver transplant recipients with Roux-en-Y gastric bypass

**DOI:** 10.3389/frtra.2026.1686667

**Published:** 2026-03-12

**Authors:** Peyton Crest, Julius S. Navarro, Fawzy Barry, Chloe Sasakado, John P. Roberts

**Affiliations:** 1 Division of Transplant, Department of Surgery, University of California, San Francisco, CA, United States; 2 Clinical Nutrition, University of California, San Francisco, CA, United States; 3 Division of Gastroenterology and Hepatology, Department of Medicine, University of California, San Francisco, CA, United States

**Keywords:** bariatric surgery, EDGE, ERCP, liver transplantation, malnutrition

## Abstract

**Introduction:**

Among liver transplant recipients, Roux-en-Y gastric bypass (RYGB) may lead to postoperative malnutrition and nutrient deficiencies. Procedures such as the endoscopic ultrasound-directed transgastric ERCP (EDGE) can result in advantageous weight gain for liver transplant recipients with prior RYGB who do not respond to enteral or parenteral nutritional interventions.

**Methods:**

We present three patients who underwent EDGE for post-transplant malnutrition and weight loss.

**Results:**

EDGE resulted in weight gain across all patients. Liver transplant recipients with a history of RYGB may be at significant nutritional risk following transplant due to fat malabsorption, underestimation of resting energy expenditure, and appetite suppression mediated by neurohormonal signals.

**Conclusion:**

When traditional nutritional interventions are unsuccessful, the EDGE procedure may be an effective alternative to promote post-transplant weight gain in this unique population.

## 1 Introduction

Obesity is one of the most prevalent diseases in the United States and is associated with significant morbidity and mortality (1, 2). Likewise, the number of obese patients undergoing liver transplantation (LT) is rising (3).

Dietary and behavioral modifications are used to promote weight loss in obese patients but have limited success. Consequently, bariatric surgery is pursued as an alternative and is considered the most effective weight loss method in the general population (4). Among transplant candidates, bariatric surgery has been shown to improve listing eligibility, with sleeve gastrectomy and Roux-en-Y gastric bypass (RYGB) being the most common procedures. Because RYGB can result in fat malabsorption, sleeve gastrectomy is preferred in LT candidates (5–7). Indeed, there are few reports of RYGB in this population, with only 48 pre-transplant RYGB cases described to date (8).

**Figure 1:**
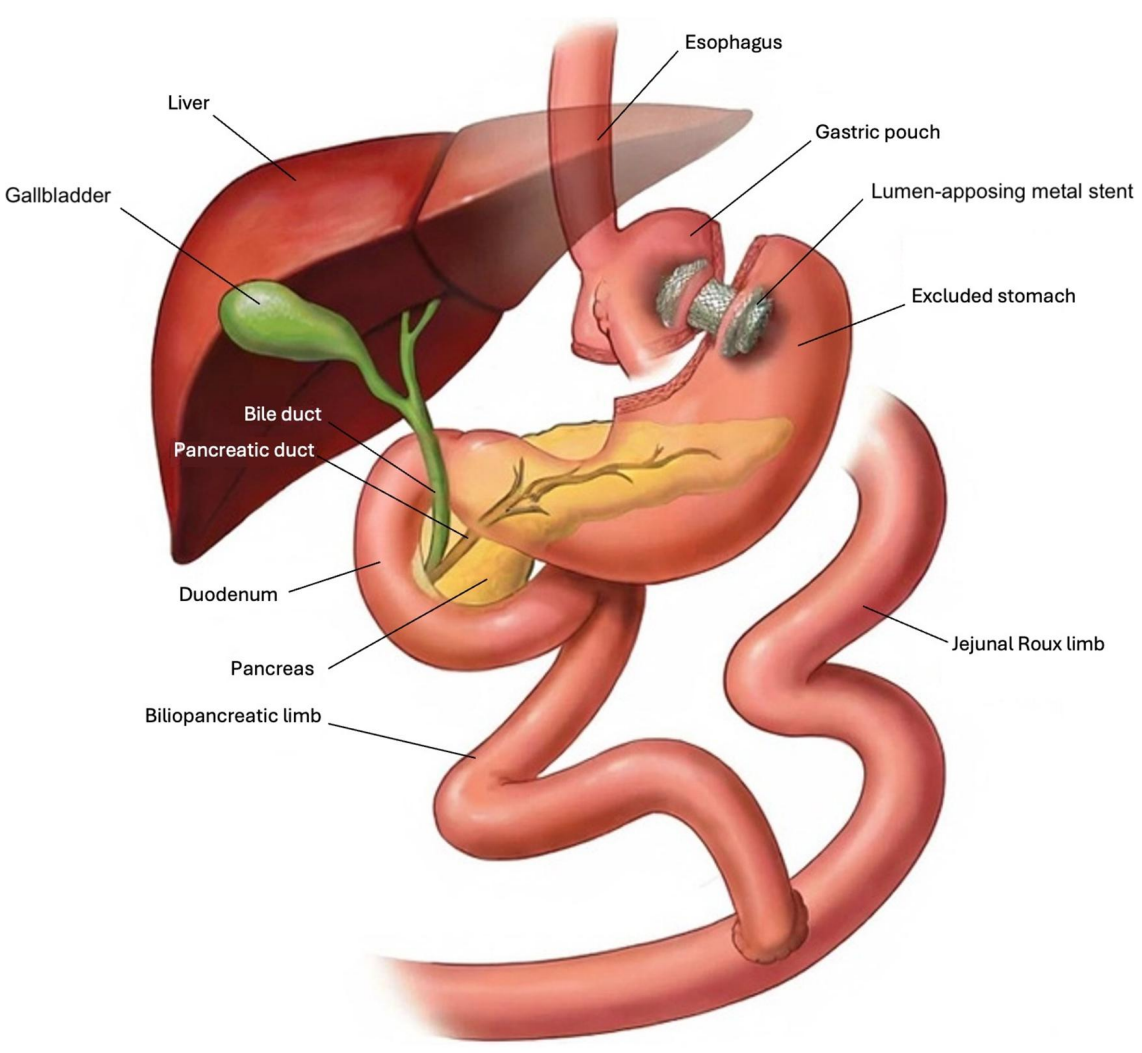
Overview of gastro-gastric endoscopic ultrasound-directed transgastric ERCP (EDGE) in Roux-en-Y gastric bypass patients. Adapted from Khara et al., Curr Gastroenterol Rep 2021;23:10 under the Creative Commons Attribution 4.0 International License (http://creativecommons.org/licenses/by/4.0/). Modifications include removal of the endoscope for clarity (11).

When RYGB patients experience post-transplant complications, procedures such as endoscopic retrograde cholangiopancreatography (ERCP) are technically challenging because a long intestinal limb separates the stomach from the sphincter of Oddi. To address these technical challenges, the endoscopic ultrasound-directed transgastric ERCP (EDGE) was developed. The EDGE procedure uses a lumen-apposing metal stent (LAMS) that connects the gastric pouch or proximal jejunum to the excluded stomach, as shown in Figure 1 (9, 10). Early reports demonstrate high technical and clinical success rates with EDGE in RYGB patients, but there is theoretical concern for procedure-related weight gain (11). While undesirable for most RYGB patients, this weight gain may be beneficial for RYGB patients diagnosed with post-transplant malnutrition.

Here we present three LT patients with a history of RYGB, who underwent EDGE to create a gastro-gastric fistula for post-transplant malnutrition.

## 2 Methods

This single-center case series screened all patients (*N* = 23) at our institution with a history of RYGB prior to LT between January 2009 and December 2024. Of these 23 patients, six required post-transplant tube feeding or total parenteral nutrition (TPN). Three patients did not undergo EDGE because they were transplanted prior to the development of EDGE (*n* = 1) or appeared well nourished on physical examination after transplant discharge (*n* = 2). In contrast, three patients underwent post-LT EDGE procedures due to persistent weight loss despite parenteral or enteral nutritional interventions. Their post-transplant nutritional status and weight trajectories were recorded.

Postoperative weight changes were made relative to body weight at post-transplant discharge to account for ascites volume and edema. Pre-transplant micronutrient deficiencies were defined as values below the normal range at our institution (Vitamin A < 38 µg/dL; Vitamin D < 30 ng/mL; Iron < 65 µg/dL; Zinc < 55 µg/dL) and were recorded if they occurred within one year of LT. Post-transplant micronutrient deficiencies were recorded if they occurred prior to EDGE, and post-EDGE micronutrient deficiencies were recorded if they occurred within one-year post-procedure. Malnutrition was defined by the criteria from the Academy of Nutrition and Dietetics (12). Estimated energy needs were calculated using the Mifflin St. Jeor equation (13), and protein needs were estimated at 1.4 g/kg/day (14). Sarcopenia was diagnosed by clinical assessment, and Liver Frailty Scores were calculated as previously described (15). This study was approved by the University of California, San Francisco Institutional Review Board (IRB #26-45868). Written informed consent was obtained from the individuals or their next of kin for the publication of any potentially identifiable data included in this article.

## 3 Case descriptions

### Case 1

A 70-year-old male with a history of RYGB underwent expedited LT secondary to HBV reactivation (Table 1). Prior to LT, he was placed on continuous renal replacement therapy (CRRT) for anuria unresponsive to diuretics.

**Table 1:** Baseline characteristics and nutritional outcomes of patients 1–3.

Characteristic	Patient 1	Patient 2	Patient 3
Time between RYGB and LT	18 years	8 years	0.5 months
Etiology	HBV	Wilson's disease	ARLD
Laboratory MELD at LT	38	36	31
Donor type; age (years)	DBD; 24	DBD; 31	DBD; 44
Pre-LT BMI (kg/m^2^)	38.2	32.9	32.9
Pre-LT micronutrient deficiencies	Vitamin A; Vitamin D; Iron	Vitamin A; Zinc	Vitamin D
Post-LT micronutrient deficiencies	None	None	Vitamin D; Iron
First malnutrition diagnosis	Pre-LT	Post-LT	Pre-LT
Osteoporosis/osteopenia diagnosis	Post-LT	—	Post-LT
Pre-LT LFI	6.76	5.68	—
Post-LT LFI	6.73	5.31^a^	—

ARLD, alcohol-related liver disease; DBD, donation after brain death; EDGE, endoscopic ultrasound-directed trans-gastric ERCP; kg, kilograms; LT, liver transplantation.

^a^ Post-EDGE Procedure.

The patient's postoperative course was complicated by diminished appetite, with the patient meeting only 23% of his caloric and 34% of his protein needs. Consequently, nocturnal tube feedings were initiated and continued after discharge.

**Figure 2:**
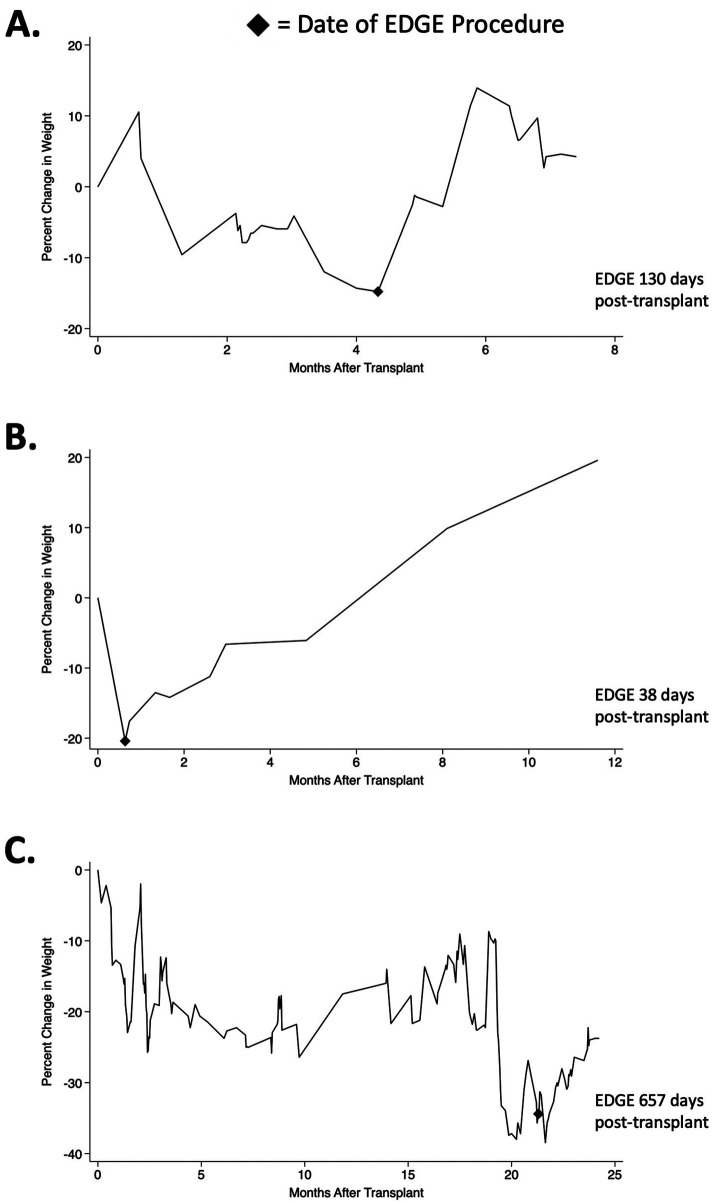
Post-transplant weight trajectories for **(A)** Patient 1, **(B)** patient 2 and **(C)** patient 3, reported as percentage of post-transplant body weight.

One day later, the patient was readmitted for surgical complications. Upon admission, oral calorie count showed that he was only meeting 21% of his caloric and 23% of his protein requirements beyond nocturnal tube feeds. He was then initiated on total parenteral nutrition (TPN) for five days and later discharged on cyclic tube feeds that provided 100% of his daily caloric and protein needs. Throughout his hospitalization, the patient lost a total of 7.9 kg [9.6% of total body weight (TBW)], as shown in Figure 2.

Two months later, the patient was readmitted with rising liver function tests. By this time, he had lost 11.8 kg since transplant (14.3% TBW), despite receiving tube feeds that met 100% of his daily caloric and protein intake. During this hospitalization, he was also diagnosed with osteoporosis and sarcopenia. Given the patient's ongoing malnutrition, an EDGE procedure was performed, creating a gastro-gastric fistula using a 20 × 10 mm LAMS. Immediately post-EDGE, the patient was started on TPN due to failure to thrive and refusal of tube feeds. He remained on TPN for 28 days before transitioning to cyclic tube feeds. Tube feeds were placed across the LAMS and delivered to the excluded stomach, meeting 100% of his caloric and protein needs. Following the patient's EDGE procedure, he gained 20.4 kg (22.3% of TBW at EDGE; Table 2). The LAMS was removed 48 days later, and the patient was subsequently discharged.

**Table 2:** Nutritional statuses and post-EDGE weight gain for patients 1–3.

Characteristic	Patient 1	Patient 2	Patient 3
Months between LT and EDGE	4	1	22
Post-EDGE micronutrient deficiencies	Iron	None	Vitamin D; Iron
Pre-EDGE weight change (%)^a^	−12.2	−20.4	−34.4
Post-EDGE weight change (%)^b^	+22.3	+17.3	+25.3

Pre-EDGE weight change represents weight at transplant discharge to last available weight pre-EDGE procedure. Post-EDGE weight change is recorded at 3 months post-procedure.

EDGE, endoscopic ultrasound-directed trans-gastric ERCP; LT, liver transplantation.

^a^ Percentage of total body weight at transplant discharge.

^b^ Percentage of total body weight at EDGE.

Forty-one days following discharge, the patient developed aspiration related to tube feeds, resulting in acute hypoxemic respiratory failure that required intubation, bronchoscopy, and supplemental oxygen. Given his severe deconditioning and multiple comorbidities, the patient was transferred to comfort care and died four months after his EDGE procedure.

### Case 2

A 47-year-old female with a history of obesity post-RYGB, hepatic encephalopathy, and esophageal varices underwent expedited LT for Wilson's disease (Table 1).

The patient's postoperative course was complicated by acute kidney injury requiring CRRT, early allograft dysfunction, coagulopathy, and malnutrition. On postoperative day nine, the patient met only 25% of her energy requirements and began cyclic tube feeds that provided 87% of her caloric and 82% of her protein needs. The patient was discharged on tube feeds, which were discontinued five days later.

Two weeks after discharge, the patient was readmitted and diagnosed with failure to thrive following a 15 kg weight loss (20.3% TBW). Given her extreme weight loss and severe muscle wasting, an EDGE procedure was performed, creating a gastro-gastric fistula using a 20 × 10 mm LAMS. Post-EDGE, she was initiated on continuous nasogastric tube feedings that were delivered to the excluded stomach. The patient's tube feedings provided 63% and 80% of her caloric and protein requirements, respectively. By discharge, three days post-EDGE, the patient had gained 2.1 kg (3.6% of TBW at EDGE) and was maintained on cyclic tube feeds.

By three months post-EDGE, the patient had gained 10.2 kg (17.3% of TBW at EDGE; Table 2), and her tube feeds had been discontinued. At present, the patient's LAMS remains in place. She continues to receive outpatient care and has demonstrated both improved nutritional status and substantial weight gain (Figure 2).

### Case 3

A 52-year-old male with a history of RYGB underwent LT for alcohol-related liver disease complicated by hepatic encephalopathy, esophageal varices, and hepatorenal syndrome (Table 1). Prior to LT, CRRT was initiated, and he remained on pre-transplant cyclic tube feeds that met 65% of his caloric needs and 68% of his protein needs to support his oral intake.

On post-transplant day six, the patient was diagnosed with malnutrition. His cyclic tube feeds were resumed, providing 89% of his caloric and 84% of his protein requirements. Despite this, by four months post-transplant, he had lost 22 kg (25.5% TBW) while continuing tube feeds.

At eighteen months post-LT, the patient underwent successful kidney transplantation. Two months later, cyclic tube feeds providing 78% of his caloric and 100% of his protein needs were initiated to address his severe malnutrition. However, the patient continued to lose weight, prompting a gastro-gastric EDGE procedure with a 20 × 10 mm LAMS. Immediately following EDGE, cyclic tube feeds were advanced to the patient's excluded stomach. The patient's tube feedings provided 65% of his caloric needs and 96% of his protein needs and were continued for one month. Three months post-EDGE, the patient gained 14.3 kg (25.3% of TBW at EDGE; Table 2), as shown in Figure 2.

Presently, the patient's LAMS remains in place. He is engaged in outpatient nutrition services and has demonstrated noticeable improvement in muscle and adipose tissue reserves.

## 4 Discussion

While previous studies have reported RYGB prior to LT (16, 17), no studies have examined nutritional management strategies and outcomes in this population. Importantly, the existing literature on the EDGE procedure has largely focused on facilitating ERCP rather than nutritional management (18); only one case to date describes its use for malnutrition in an LT recipient with prior RYGB (19). Here, we report three LT recipients with a history of RYGB who demonstrated significant and prolonged post-transplant malnutrition, which was managed with the EDGE procedure.

Cachexia, malnutrition, frailty, and sarcopenia are well-recognized consequences of end-stage liver disease (20). Post-transplant, energy needs are heightened, and nutritional status can deteriorate rapidly, increasing patients’ risk of malnutrition (21). Adequate nutritional support is therefore essential to prevent negative energy balance and subsequent weight loss, which are associated with significant post-transplant morbidity and mortality (22, 23). Across our three cases, the clinical impact of persistent malnutrition was profound: all patients had micronutrient deficiencies (Table 1) and two were diagnosed with osteopenia/osteoporosis (Cases 1 and 3), sarcopenia (Cases 1 and 2), and frailty (Cases 1 and 2).

Previous data suggest that nutritional interventions are necessary to ameliorate post-transplant malnutrition (21). Specifically, early postoperative enteral or parenteral nutrition is crucial in improving nitrogen balance and promoting recovery (24, 25). While these interventions are effective in LT recipients without a history of RYGB, we observed persistent weight loss in our cohort despite administration of enteral or parenteral nutrition.

This persistent weight loss may be explained by three interrelated factors: (1) malabsorption of fat after RYGB, (2) underestimation of RYGB resting energy expenditure (REE) in the post-transplant setting, and (3) appetite suppression mediated by neurohormonal signals after RYGB. First, in RYGB patients, bypassing the duodenum impairs macro- and micronutrient absorption in the proximal small intestine and prevents mixing of ingested nutrients with bile acids and pancreatic enzymes (26). Consequently, LT recipients with RYGB, especially those with longer Roux limbs, may be predisposed to malabsorption, which can hinder post-transplant hepatocyte recovery (27). Of particular concern is fat malabsorption; the coefficient of fat absorption decreases from 92%–95% pre-operatively to approximately 68%–75% post-RYGB, corresponding to a daily fecal fat loss of 10–12 g (28).

Furthermore, there is concern about the accuracy of REE estimations for LT recipients with RYGB. While indirect calorimetry remains the gold standard for accurate post-transplant REE calculations, REE is often estimated using predictive equations, such as the Harris-Benedict (29) or Mifflin-St. Jeor equations (13). These equations, however, were developed in healthy subjects and cannot account for the metabolic changes that occur after transplantation. For example, a systematic review of 1,883 cirrhotic patients revealed that 83% of predictive equations underestimated the energy needs of patients when compared with indirect calorimetry (30). Similarly, a prospective observational study found that predictive equations, such as the rule of thumb method, Harris-Benedict, Ireton-Jones, and Penn State, exhibit significant individual variation in REE among LT recipients (31, 32). These discrepancies may be even more pronounced for LT recipients with prior RYGB, as RYGB has been shown to increase the thermogenic effect of food (33, 34). Consequently, LT recipients with RYGB may have substantial REE requirements that are not adequately captured by traditional REE models, contributing to inadequate caloric delivery.

Lastly, appetite suppression, likely mediated by neurohormonal signals, is the primary driver of weight loss after RYGB. This mechanism may significantly contribute to the early satiety and nutritional difficulties that arise following LT (35).

The above factors suggest that interventions other than increased nutritional supplementation (e.g., tube feedings) may be effective in LT recipients with RYGB. As illustrated in Figure 2, all patients demonstrated significant weight loss prior to EDGE intervention. Post-EDGE, however, all experienced weight gain, with two of three patients reaching their baseline weight within one year post-transplant (Figure 2). In contrast, among the three patients at our center who received post-transplant tube feedings without EDGE, only one reached their baseline weight within one year post-transplant. While other data have demonstrated variable weight changes in standard RYGB populations following EDGE (36), the combination of severe pre-EDGE malnutrition and intensive enteral or parenteral support may explain the pronounced weight gain we observed.

Although the relative contribution of restored gastric continuity vs. increased caloric intake remains unclear, previous studies have reported weight gain among RYGB with gastro-gastric fistulas (37–39). Additionally, Cases 2 and 3 showed increased daily calorie counts compared to those prior to EDGE (Case 2: 2,248 kcal vs. 2,643 kcal; Case 3: 754 kcal vs. 2,400 kcal), and Case 1 received higher-calorie parenteral feedings post-EDGE (1,560 kcal vs. 2,346 kcal). Increased tube feedings, particularly high-protein formulations, may be poorly tolerated in RYGB patients (40, 41) but may improve following EDGE. Thus, post-EDGE weight gain likely reflects restored gastric continuity and improved feeding tolerance.

This case series is the largest study to date that explores the EDGE procedure in LT recipients presenting with impaired nutritional status. Our three cases collectively demonstrate that LT recipients may encounter greater postoperative nutritional challenges if they have a history of RYGB. This is exacerbated by the reliance on predictive REE equations, which fail to account for the increased energy demands of this population, leading to persistent negative energy balance and clinical deterioration despite aggressive enteral or parenteral support. The EDGE procedure, in turn, may address these challenges and lead to meaningful weight gain in this population.

Several limitations must be considered when interpreting our findings, including our small sample size, absence of a control group, and use of predictive equations to estimate REE. All patients had complex nutritional needs post-transplant, and a variety of methods were used to facilitate weight gain post-EDGE (TPN, continuous tube feeding, cyclic feeds). Whether weight gain can be strictly attributed to EDGE is hard to determine given the small number of patients in our sample. Moreover, the length of patients’ Roux limbs was not available, which has been associated with malabsorption and malnutrition risk (27). This limits our ability to determine which patients may experience greater weight gain following EDGE.

Our study provides insight into the postoperative management of LT recipients with a history of RYGB. When tube feedings are unsuccessful among these patients, the EDGE procedure may be an effective intervention, especially in the context of increased REE and macro- and micronutrient malabsorption. Further research is needed to determine energy requirements, the effectiveness of the EDGE procedure, and optimal timing for post-transplant nutritional interventions in this unique population.

## Data Availability

The data analyzed in this study is subject to the following licenses/restrictions: The datasets for this article are not publicly available due to concerns regarding participant/patient anonymity. Requests to access these datasets should be directed to john.roberts@ucsf.edu.
